# Think small

**DOI:** 10.7554/eLife.22497

**Published:** 2016-12-06

**Authors:** Luis A Bezares-Calderón, Gáspár Jékely

**Affiliations:** Max-Planck-Institute for Developmental Biology, Tübingen, Germany; Max-Planck-Institute for Developmental Biology, Tübingen, Germanygaspar.jekely@tuebingen.mpg.de

**Keywords:** connectome, synapse, network, CNS, central nervous system, *C. intestinalis*

## Abstract

The tadpole larva of a sea squirt is only the second animal to have its entire nervous system mapped out, and the results confirm that there is still much to learn from the smallest brains.

**Related research article** Ryan K, Lu Z, Meinertzhagen IA. 2016. The CNS connectome of a tadpole larva of *Ciona intestinalis* (L.) highlights sidedness in the brain of a chordate sibling. *eLife* **5**:e16962. doi: 10.7554/eLife.16962

Brains come in different sizes. Sperm whales have the largest, weighing in at over 7 kilograms, whereas the dwarf males of a worm called *Dinophilus gyrociliatus* have the smallest, containing just 66 neurons (Windoffer and Westheide, 1988). Whether large or small, one feature unites all brains more than anything else: their neurons are connected to form intricate networks. This property is not seen in other organs, like the liver, kidneys or skin. Mapping networks of neurons has now become a central theme in neuroscience, as evidenced by the rise of the field of connectomics.

Producing wiring diagrams of neural networks – or connectomes – that show the specific connections between cells requires use of electron microscopy to image ultrathin slices of neural tissue. This approach has led to spectacular progress in recent years, but it can only analyse relatively small volumes. For example, a mouse brain is approximately 1 cm^3^, which is about 100,000 times larger than the volume that can currently be imaged and analysed. This is why size matters in connectomics. To date, a complete connectome has been produced for just one species, the nematode worm *Caenorhabditis elegans*. This wiring diagram, which was published 30 years ago, contains 302 neurons and took over a decade of effort (White et al., 1986).

Now, in eLife, Kerrianne Ryan, Zhiyuan Lu and Ian Meinertzhagen from Dalhousie University report that they have mapped the connectome of a tadpole larva of the ascidian *Ciona intestinalis* (Ryan et al., 2016). Adult ascidians are quite unremarkable, water-filled sacs that squirt when removed from the sea – which is why they are commonly called “sea squirts” (Figure 1A). However, their tadpole-like larvae have attracted interest ever since the Russian embryologist Alexander Kowalewsky discovered in 1867 that they have the same basic body plan as the vertebrates (i.e. animals with backbones, ranging from fish to humans; Mikhailov and Gilbert, 2002). Later molecular studies confirmed that they were indeed our closest “invertebrate” relatives (Delsuc et al., 2006), and *Ciona* has now become an important laboratory animal in many areas of biology.

**Figure 1.**
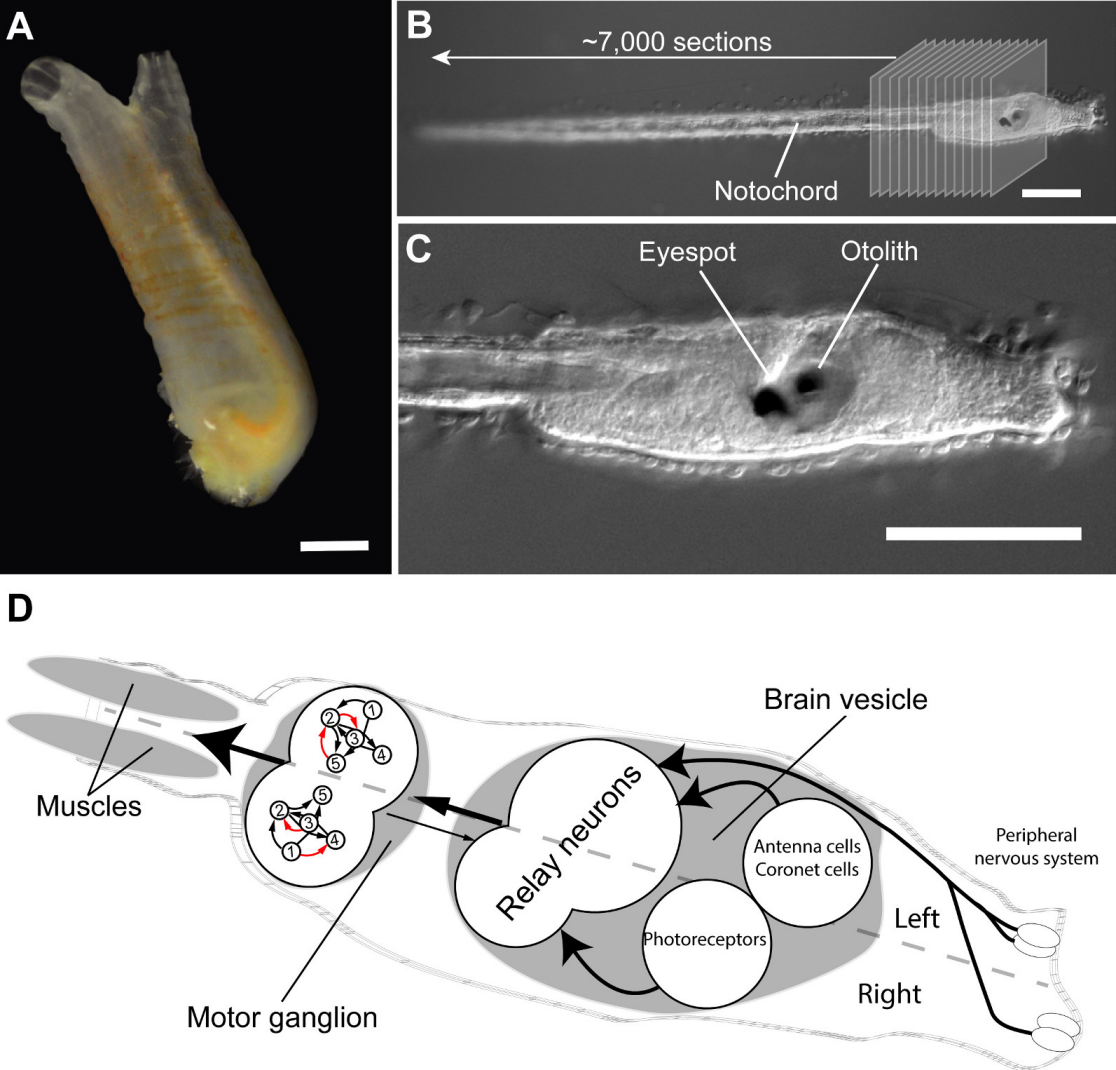
Connectome of the *Ciona intestinalis tadpole* larva. (**A**) As adults, sea squirts like *Ciona intestinalis* are immobile, filter-feeding animals. The scale bar is 10 mm. (**B**) The tadpole-like larva of *Ciona*, however, can swim and has a vertebrate-like body plan with a flexible rod that runs along its back called a notochord. Ryan et al. cut a single larva into ~7,000 ultrathin slices and used these to reconstruct the larval connectome. (**C**) The larva also has sensory organs, including a single eyespot on the right side and an organ involved in sensing gravity (called otolith) on the left. The scale bars in B and C are 100 μm. (**D**) The *Ciona* connectome shows that the larval nervous system consists of a peripheral and a central nervous system; the latter includes a brain vesicle and a motor ganglion. The brain vesicle contains the photoreceptors for the eyespot, the coronet cells (which are thought to detect pressure), antenna cells (which are involved in sensing the position of the otolith), and relay neurons. The motor ganglion contains neurons that directly control the contractions of the muscles. The brain shows many asymmetries in its cellular anatomy and wiring. For example, there are more relay neurons on the left of the larvae, and the motorneurons in the motor ganglion (numbered 1–5) are connected differently on the left and right sides of the larvae (red arrows show asymmetric connections). Photographs in A, B and C are courtesy of Antonio Palladino.

With fewer than 200 neurons, the nervous system of a *Ciona* larva is small and comes close to the lower limits of brain size. Nevertheless, imaging and reconstructing most of the neurons in the nervous system of a single specimen required a gargantuan effort. First, Ryan et al. sliced a single larva into 7,000 sections, just tens of nanometres thick, and imaged the sections using electron microscopy (Figure 1B). The high-resolution images were then aligned and the neurons were painstakingly traced by hand. Finally, the connections for each and every neuron were identified and annotated. This resulted in the near-complete wiring diagram of the *Ciona* larva, representing the second connectome of any animal.

So, what can we learn from the connectome of *Ciona*? First, it is what is called a “small-world” network, meaning that it has highly connected local sub-networks linked together by a smaller number of long-range connections. This is similar to how the *C. elegans* connectome, social networks and power grids are organised (Watts and Strogatz, 1998). Overall, the 177 neurons are connected such that, on average, it only takes 2.7 steps to go from one node in the network to another.

The connectome also shows that a *Ciona* larva has clearly separate peripheral and central nervous systems. The peripheral nervous system contains a few sensory neurons that are in direct contact with the environment. The central nervous system consists of sensory systems containing a single eye on the right side of the head, an organ that is sensitive to gravity, and cells that are thought to sense pressure (Figure 1C). These sensory systems connect to highly interconnected relay neurons found at the back of the brain. These relay neurons in turn project to interneurons and motorneurons that are also part of the central nervous system and that eventually innervate into the larva’s muscles.

How can the connectome help us understand how the *Ciona* larva does what it does? Well, almost everything these creatures do involves their muscles. Even before hatching, asymmetric muscle contractions cause the larva’s tail to flick 10 times per second and help it to hatch from its egg. Tail flicks then persist after hatching and may be important for changing direction when swimming (Mast, 1921). Moreover, as a larva ages, its movements change and become driven by symmetric tail contractions that occur about 20–30 times per second. The larvae also respond to different stimuli – including light, gravity and shadows – by altering the activity of their muscles (Zega et al., 2006; McHenry and Strother, 2003); for example, young larvae will swim up against the direction of gravity (Tsuda et al., 2003). Ryan et al. have now fully mapped neuronal pathways responsible for these relatively simple behaviours, from sensory organs to muscles (Figure 1D).

Simple as these behaviours may be, they are not independent. Instead, the network allows crosstalk at different levels. This suggests that the brain may indeed integrate different stimuli but only trigger one pattern of movements at a time – which is consistent with how these larvae actually swim (Zega et al., 2006). These findings show that, even though each larva is small and anatomically simple, the underlying neural networks in its nervous system are complex and integrated.

Ryan et al. noted another intriguing feature of the *Ciona* connectome. Most animals studied to date display a left-right symmetry, so that for each neuron on the left, there is a counterpart on the right. However, most of the nervous system of *Ciona* lacks this symmetry (Figure 1D). Moreover, even those groups of neurons that are symmetric – such as the motorneurons – are connected to other cells in an asymmetric way. It is not known why the nervous system of *Ciona* is so strongly asymmetric, but it may have something to do with the streamlining of the ascidian body plan. Asymmetries can be found in all brains, including our own, but to a lesser extent; so *Ciona* may provide clues on the development and function of asymmetry in brains.

The *Ciona* connectome holds lessons for those trying to understand how the brain works. It clearly shows that we are not done with the smallest brains yet; despite having few neurons, the *Ciona* network is complex and we are still far from fully understanding it. More generally, it suggests that it would be wise to solve some other small brains before we tackle more complex ones. This suggestion also makes economic sense, when you consider that a single mouse connectome would roughly cost the same as 100,000 small brain connectomes. Perhaps others working in the connectomics field will be inspired by the beautiful work of Ryan, Lu and Meinertzhagen, and turn their attention to the smallest of brains first.
